# Is higher lymphocyte count a potential strategy for preventing chronic kidney disease in patients receiving long-term dasatinib treatment?

**DOI:** 10.1186/s40780-022-00270-x

**Published:** 2023-01-23

**Authors:** Hirokazu Nakayama, Hiromitsu Iizuka, Toshiaki Kato, Kensuke Usuki

**Affiliations:** 1 Department of Pharmacy, NTT Medical Center Tokyo, 5-9-22 Higashi-Gotanda, Shinagawa-Ku, Tokyo, 141-8625 Japan; 2 Department of Hematology, NTT Medical Center Tokyo, 5-9-22 Higashi-Gotanda, Shinagawa-Ku, Tokyo, 141-8625 Japan

**Keywords:** Dasatinib, Chronic kidney disease, Lymphocyte count, Neutrophil–lymphocyte count ratio, Philadelphia chromosome positive acute lymphoblastic leukemia and chronic myeloid leukemia

## Abstract

**Background:**

Dasatinib, which is used to treat treating chronic myeloid leukemia, induces increases in blood lymphocytes during the treatment. In addition, neutrophil–lymphocyte count ratio (NLR) is associated with the related to development of chronic kidney disease (CKD). However, it has not been reported whether development of CKD during long-term dasatinib treatment is related to lymphocyte count or NLR. This study aimed to reveal the relationship between CKD and lymphocyte count or NLR during long-term dasatinib treatment.

**Methods:**

A retrospective study was conducted in patients treated with dasatinib for 6 months or longer. Risk factors for CKD development were explored using multivariate analysis. Changes in maximal lymphocyte count and NLR over time were examined separately.

**Results:**

A total of 33 patients in CKD group (*n* = 8) and No CKD group (*n* = 25) who received dasatinib were enrolled. In univariate analysis, significant differences between the groups were observed in maximal lymphocyte count, lymphocytosis, age, and estimated glomerular filtration rate at baseline. As the factor independently associated with the development of CKD, maximal lymphocyte count (odds ratio 0.999, 95% confidence interval: 0.999–1.000, *p* = 0.033) was identified. In this analysis, age had borderline significance (odds ratio 1.073, 95% CI: 0.999–1.153, *p* = 0.054)]. After 6 months of dasatinib therapy, lymphocyte count was significantly lower in CKD group [median (range), 2184 (878‒3444)/μL] than in the No CKD group [3501 (966‒7888)/μL] (*p* = 0.020). However, no significant difference in lymphocyte count was observed between the groups at the last follow-up. During the study period, the median NLR in the No CKD group fluctuated between 1.11 and 1.42, and median NLR in CKD group was increased from 1.13 to 2.24 between after 6 months of dasatinib therapy and the last follow-up.

**Conclusions:**

The development of CKD during dasatinib therapy was associated with lower maximal lymphocyte counts. In contrast, the higher levels of lymphocytes induced during dasatinib treatment may prevent CKD progression.

## Background

Dasatinib, a targeted inhibitor of oncogenic breakpoint cluster region-Abelson leukemia virus (BCR-ABL) tyrosine kinase, has profoundly improved the outcome of chronic myeloid leukemia (CML) and Philadelphia-chromosome-positive acute lymphoblastic leukemia (Ph-ALL). In patients with CML, near-normal life expectancy is possible by treatment with dasatinib as first-line therapy or as second-line treatment in patients intolerant or resistant to other tyrosine kinase inhibitors (TKIs) [1–3]. In patients treated with TKI, such as imatinib, age and co-morbidities of hypertension and diabetes mellitus have been identified as risk factors of chronic kidney disease (CKD) [4]. In addition, some studies have reported the development nephrotic syndrome or proteinuria in patients treated with dasatinib [5, 6].

Since neutrophil–lymphocyte count ratio (NLR) has been associated with inflammatory status, higher NLR is a potential indicator of deterioration of kidney function in patients with CKD [7]. In addition, since dasatinib induces increases in blood lymphocytes and monocytes with modest reduction in the relative proportions of neutrophils and eosinophils after 4 weeks of treatment, at least approximately 50% patients developed lymphocytosis which is associated with increase of overall survival [8]. However, it has not been reported whether development of CKD during long-term dasatinib treatment is related to lymphocyte count or NLR.

The objectives of this study were to reveal whether increases in lymphocytes and/or reduction of NLR is associated with development of CKD in patients on long-term dasatinib treatment.

## Methods

### Study design and population

The present study was a secondary analysis based on the data collected in our previous study that examined the risk factors of creatine kinase elevation during dasatinib treatment [9]. A retrospective medical chart review was conducted at NTT Medical Center Tokyo. Patients initiated dasatinib treatment between March 16, 2009 and March 31, 2019 were enrolled. The subjects eligible for this study were patients aged 20 years or older with a diagnosis of CML or Ph-ALL, who were treated with dasatinib for 6 months or longer. Patients who developed CKD at baseline were excluded from the study. Primary outcome was development of CKD. This chart review study was approved by the Ethics Committee of NTT Medical Center, Tokyo (approval number: 20 − 107) prior to study commencement. In addition, a waiver of written informed consent was granted due to the retrospective nature.

### Data collection

The following data were retrieved from electronic medical records between baseline and the last follow-up as long as the patient remained in the present study: demographics; primary diagnosis; complete blood count; blood chemistry including albumin at baseline; kidney function test of creatinine and estimated glomerular filtration rate (eGFR); presence of CKD at baseline; concomitant use of known nephrotoxic medications (including aminoglycosides, vancomycin, diuretics, angiotensin-converting enzyme inhibitors, angiotensin receptor blockers, and non-steroid anti-inflammatory drugs); previous use of other TKIs; development of CKD during the periods in patients received other TKIs prior to dasatinib; lymphocytosis; and onset of adverse events. In addition, the ratio of maximal to baseline lymphocyte count was calculated. In the present study, GFR was estimated using the Modification of Diet in Renal Disease equation that has been shown to provide a more accurate estimation in Japanese [10]. In patients with CML, Sokal score was calculated using age, platelet count, myeloblast count (%) and spleen size (maximum distance palpable below the costal margin) at diagnosis, according to a previous report [11].

### Study period

In the analysis of development of adverse events, lymphocytosis, lymphocyte counts, and NLR, the study period was defined as the duration from after 4 weeks (4-weeks) of dasatinib treatment to the last follow-up for patients who did not develop CKD. For patients who developed CKD, the duration from after 4 weeks of dasatinib therapy to the onset of CKD was included.

### Follow-up

A complete blood count was performed at least once a week for the first 1 month, and once every 1 to 12 weeks thereafter. In all patients, chest X-ray was performed prior to dasatinib treatment and during treatment. While patients were administered a standard dose of dasatinib, initial and maintenance dosages with modification were considered in special populations, such as elderly individuals. Follow-up interval, time of chest X-ray examination, and interruption or dosage reduction of dasatinib therapy due to adverse events were decided at the discretion of the treating physician.

### Definition of CKD

Since serum creatinine fluctuates greatly, median serum creatinine level was calculated for 7 days before starting dasatinib therapy, and the median level was defined as baseline serum creatinine. Development of CKD was defined as an estimated eGFR less than 60 mL/min/1.73 m^2^ persisting for 90 days or longer. In patients treated with other TKIs prior to dasatinib, development of CKD during the periods in which other TKIs were administered was examined separately.

### Longitudinal analysis

In the analysis between development of CKD and lymphocyte count or NLR, changes in the maximal lymphocyte count and NLR over time were separately examined at baseline, 4-weeks, after 6 months (6-months), and at the last follow-up of dasatinib treatment. NLR was obtained by dividing the absolute blood neutrophil count by the lymphocyte count for each complete blood count monitoring during follow-up. Median lymphocyte count and NLR were calculated.

### Lymphocytosis

Lymphocytosis during dasatinib treatment was defined as an absolute blood lymphocyte count > 3.6 × 10^9^/L on 2 or more occasions after 4 weeks of dasatinib therapy, according to previous reports [12, 13].

### Definition of adverse event

In the present study, pleural effusion, colitis and pulmonary hypertension were defined as common dasatinib-related adverse events. Pleural effusion and colitis at grade 2 or higher were defined as adverse events, according to the Common Terminology Criteria for Adverse Events version 4.03 [14]. Development of pulmonary hypertension diagnosed by a cardiologist based on comprehensive assessments including chest X-ray and transthoracic echocardiography was defined as adverse event.

### Statistical analysis

The subjects were classified into CKD group and No CKD group. All data were expressed as median (range). Statistical comparison of relevant clinical data between two groups was performed using Mann–Whitney U test. Chi-square test or Fisher exact test was used to compare categorical data between groups. Univariate analysis was conducted to screen covariates, and variables with collinearity and *p* values less than 0.05 were considered eligible for multivariate analysis. For multivariate analysis, logistic regression analysis was used to identify the risk factors for CKD using a stepwise method. Correlation between maximal or minimal lymphocyte count and median lymphocyte count during the study period was examined using Spearman’s rank correlation test. A *p* value less than 0.05 was considered statistically significant. Statistical analyses were performed using IBM SPSS Statistics version 24 (IBM Japan, Tokyo, Japan).

## Results

A total of 64 patients received dasatinib treatment. Among them, 31 patients were excluded for the following reasons‒dasatinib treatment duration less than 6 months (*n* = 24), no available baseline data due to referral to our medical center from another institute (*n* = 4), and development of CKD at baseline (*n* = 3). Eventually, 33 patients (25 men and 11 women), comprised 30 CML patients and 3 Ph-ALL patients, were enrolled. Their median (range) age was 45 (29‒86) years and eGFR was 77 (55‒103) mL/min/1.73 m^2^ (CKD stage 1 in 4 and stage 2 in 29 patients). The follow-up duration was 27 (6‒100) months. The patients received initial doses of dasatinib ranging from 20 to 140 mg per day, once daily or divided twice daily. In patients with CML, Sokal risk score at diagnosis was “low” in 20, “intermediate” in 6 and “high” in 3 patients, and not available in 1 patient. Prior to dasatinib treatment, 20 patients received other TKIs: imatinib in 11, nilotinib in 4, and both in 5 patients. In those 20 patients, the median age at the initiation of other TKIs prior to dasatinib treatment was 40 years, and no development of CKD was observed at the duration of administration of other TKIs.

During the follow-up, 8 patients developed new-onset CKD with deterioration of CKD stage 2 to 3a and were classified into the CKD group, and 25 patients were classified into the No CKD group. The dasatinib dose was not reduced in any of the patients who had onset of CKD. In the univariate analysis, the CKD group had significantly higher age [60 (36‒86) years], lower eGFR [68 (61‒77) mL/min/1.73m^2^] at baseline and lower maximum lymphocyte count [3279 (1246‒4183) (/µ/L)] during the examined period, compared to the No CKD group. In addition, 17 patients (68%) without CKD developed lymphocytosis, the rate of which was significantly higher than that of patients with CKD (*p* = 0.012). The two groups did not differ significantly in sex ratio, serum albumin, creatinine, development of adverse events, concomitant nephrotoxic agents, hypertension, diabetes mellitus, previous use of other TKIs, Sokal score, the ratio of maximal-baseline lymphocyte count and NLR. However, a significantly higher frequency of lymphocytosis was observed in No CKD group than in CKD group (*p* = 0.018) (Table 1).

**Table 1:** Univariate analysis of patient characteristics between the two group

Variable	CKD Group (*n* = 8)	No CKD Group (*n* = 25)	*p*-value
Age (years)	**60 (36–86)**	**43 (29–70)**	**0.008**
Sex (male/female)	**5/3**	**17/8**	**1.000**
CML/Ph-ALL	**6/2**	**24/1**	**0.139**
Serum creatinine level (mg/dL) at baseline	**0.93 (0.55–1.02)**	**0.78 (0.53–1.12)**	**0.435**
eGFR (mL/min/1.73 m^2^) at baseline	**68 (61–77)**	**80 (55–103)**	**0.003**
Hypertension	**2**	**2**	**0.062**
Diabetes mellitus	**0**	**4**	**1.000**
Common dasatinib-related adverse events^a)^	**5**	**13**	**1.000**
Pleural effusion	**3**	**7**	**0.623**
Lymphocytosis	**1**	**17**	**0.012**
Lymphopenia	**2**	**3**	**0.574**
Concomitant potential nephrotoxic agents	**3**	**6**	**0.651**
Concomitant use of diuretics	**3**	**8**	**0.420**
Median lymphocyte count (/μL)	**2025 (780–3413)**	**2760 (1089–6200)**	**0.089**
Maximal lymphocyte count (/μL)	**3279 (1246–4183)**	**4813 (2075–10,545)**	**0.001**
Minimum lymphocyte count (/μL)	**896 (390–2136)**	**1184 (484‒2288)**	**0.127**
Maximal-baseline lymphocyte count ratio	**1.5 (1.0–3.8)**	**2.2 (0.8–9.3)**	**0.176**
Median NLR	**1.59 (0.75–4.58)**	**1.21 (0.40–2.22)**	**0.136**
History of TKI(s) prior to dasatinib	**6**	**14**	**0.431**
(Imatinib/nilotinib/Both)	**(3/1/2)**	**(8/3/3)**	

*Abbreviations*: *CKD* chronic kidney disease, *CML* chronic myeloid leukemia, *eGFR* estimated glomerular filtration rate, *NLR* neutrophil–lymphocyte count ratio, *Ph-ALL* Philadelphia chromosome-positive acute lymphoblastic leukemia, *TKI* tyrosine kinase inhibitor

^1^Pleural effusion, colitis, and pulmonary hypertension were included as common dasatinib-related adverse events

From the univariate analysis, variables with *p* values less than 0.05 (age, baseline eGFR and maximum lymphocyte count) were entered into multivariate analysis. Lymphocytosis was not entered into the multivariate model due to its collinearity with maximal lymphocyte count. In multivariate logistic regression analysis, maximal lymphocyte count was identified as a factor independently associated with the development of CKD (odds ratio 0.999, 95% CI 0.999–1.000, *p* = 0.033). Age was found to be borderline significant (odds ratio 1.073, 95% CI 0.999–1.153, *p* = 0.054) (Table 2).

**Table 2:** Multivariate logistic regression analysis of the risk factors for chronic kidney disease

Variable	Odds ratio (95% CI)	*p*-value
Age (years)	**1.073 (0.999–1.153)**	**0.054**
Maximal lymphocyte count (/μL)	**0.999 (0.999–1.000)**	**0.033**

*Abbreviation*: *CI* confidence interval

**Fig. 1:**
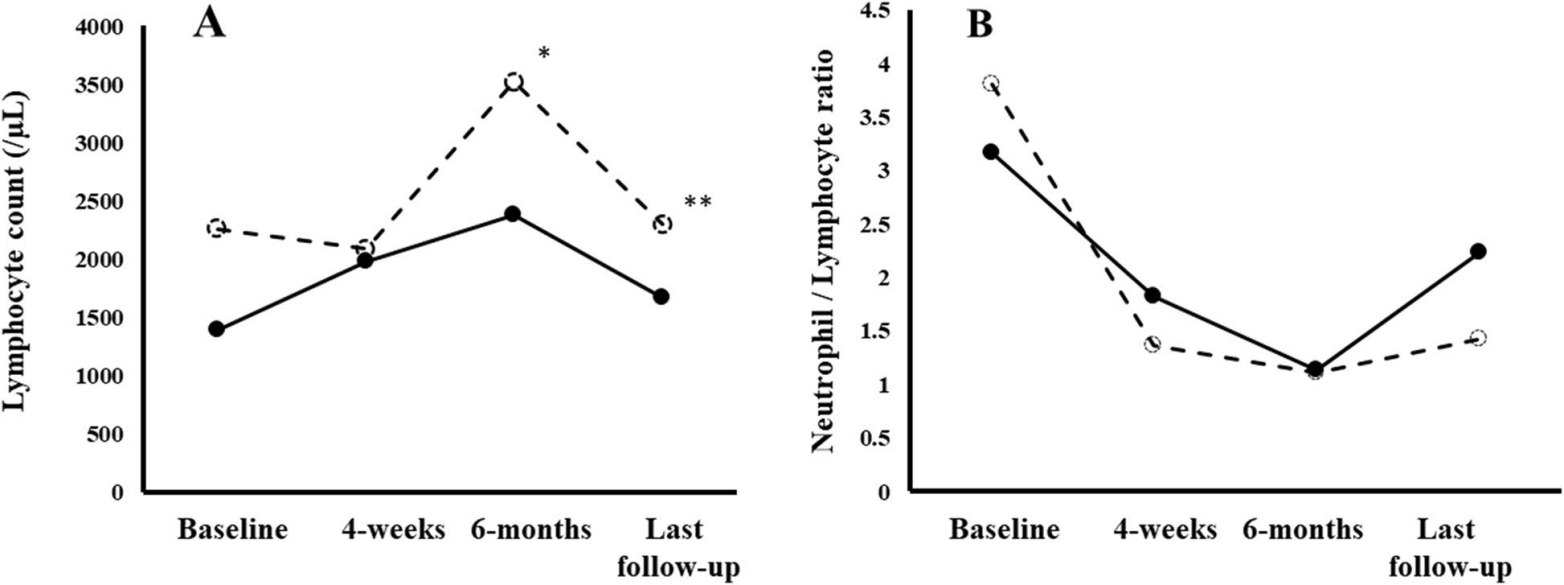
Changes in the lymphocyte count (**A**) and neutrophil–lymphocyte count ratio (**B**) at baseline, after 4 weeks of dasatinib therapy, after 6 months of dasatinib therapy, and at the last follow-up. The follow-up durations were 12 (6‒76) months in the chronic kidney disease (CKD) group and 28 (7‒100) months in No CKD group, respectively. Solid circles with a solid line indicate the CKD group. Open circles with a dashed line indicate the No CKD group. *Difference between the groups, *p* = 0.020. ** Difference between the groups, *p* = 0.098

Lymphocyte count increased between 4-weeks and 6-months of dasatnib therapy. Thereafter, lymphocyte count decreased at the last follow-up. At 6-months, lymphocyte count was significantly lower in CKD group [2184 (878‒3444)/μL] than in the No CKD group [3501 (966‒7888)/μL) (*p* = 0.020). However, no significant difference was observed between the groups at the last follow-up (Fig. 1A).

In addition, while median value of NLR fluctuated between 1.11 and 1.42 in No CKD group, that in CKD group increased from 1.13 at 6 months after dasatinib therapy to 2.24 at the last follow-up. No significant difference was observed in the NLR at 4-weeks, 6-months, and the last follow-up between the groups (Fig. 1B).

There was a strong and significant rank correlation between maximal and median lymphocyte counts (*r* = 0.772, *p* < 0.001).

## Discussion

This Japanese single center study retrospectively evaluated dasatinib-associated the development of CKD in CML or Ph-ALL patients treated with dasatinib 6 months or longer. We found that 24% of the subjects had newly developed CKD. The development of CKD during long-term dasatinib therapy was associated with lower maximal lymphocyte count, regardless of significant differences in age and eGFR at baseline in univariate analysis. In the present study, there were no significant differences between the groups in the frequency of dasatinib-related adverse associated with lymphocytosis as well as the ratio of maximal-baseline lymphocyte count. In addition, maximal lymphocyte count was strongly correlated with median lymphocyte count during the study period. Furthermore, a significant increase in lymphocyte count at 6 months after dasatinib therapy was observed between the groups. In addition, lower value of NLR was continued between 1.11 and 1.42 in No CKD group.

Caution should be exercised to generalize our findings. The eGFR at baseline was significantly lower in CKD group than in the No CKD group. Because of the small number of patients with CKD development, 2 covariates, the maximal lymphocyte count and age, were identified in multivariate analysis using the stepwise method. Eventually, eGFR at baseline was not excluded as a risk factor for CKD. Furthermore, median decrease in the eGFR between baseline and the last follow-up was 15% in CKD group. In this analysis, a subtle reduction was included due to the assessment of deterioration of CKD stage from 2 to 3a. In addition, the frequency of CKD occurrence was higher in this analysis (24%) than that in previous reports (5‒7%) [4, 15]. In this regard, the median eGFR at baseline was lower (77 mL/min/1.73 m^2^) in this study than in previous reports (87 and 95 mL/min/1.73 m^2^, respectively) in patients without CKD. Furthermore, 88% of 33 patients in this study had baseline CKD stage 2. Therefore, the difference in baseline kidney function may be associated with the higher rate of CKD development herein. Nevertheless, maximal lymphocyte count was identified rather than eGFR at baseline.

In general, since kidney structure and function are associated with age-related changes, eGFR decreases with advancing age. However, in our study, lower maximal lymphocyte count was an additional risk factor for CKD. In contrast, a higher lymphocyte count may prevent CKD progression. In this regard, our findings are consistent with those of a previous report [15].

Since NLR has been associated with inflammatory status, NLR increases with CKD stage deterioration [7]. In addition, since dasatinib induces increases in blood lymphocytes after 4 weeks of treatment [8], a higher lymphocyte count in dasatinib treatment may lead to a reduced NLR. Indeed, median NLR in No CKD group continued to be lower. Despite the shorter follow-up duration in CKD group, NLR increased from 1.13 to 2.24 between 6-months and the last follow-up (Fig. 1B). The NLR of 1.87 or higher was associated with deterioration of kidney function [16]. Thus, continuation of lower NLR in No CKD group may be a possible explanation for the prevention of CKD development in the present study. However, due to the large variability in lymphocyte count and NLR fluctuation during dasatinib treatment, it would be difficult to assess this possibility in a small-scale study.

Herein, 2 patients, an 86-year-old woman and a 70-year-old woman, received dasatinib at doses of 20 mg and 50 mg daily, respectively. While the patient who received dasatinib at a dose of 50 mg daily did not develop CKD, the other patient who received dasatinib at a dose of 20 mg daily developed CKD. Recently, Naqvi and colleagues reported that during long-term follow-up, low-dose dasatinib (50 mg daily) remained effective and safe in patients with newly diagnosed chronic-phase CML [17]. In the management of elderly patients with myeloproliferative neoplasms, justification and tolerance for treatment associated with cancer-independent life expectancy are troublesome [18]. Particularly, in elderly patients with CML treated with dasatinib, low-dose therapy (20 mg daily) leads to an adequate molecular response without causing severe adverse events [19]. Therefore, careful monitoring of kidney function is required for patients treated with low-dose dasatinib.

The molecular mechanisms underlying CKD in patients treated with dasatinib have not yet been sufficiently elucidated. However, since dasatinib is involved with the platelet-derived growth factor and Src family kinases other than BCR-ABL, a possible mechanism of nephrotoxicity during dasatinib treatment is the disruption of the vascular endothelial growth factor signalling pathway through inhibition of the Src family kinases [5, 20].

The present study has several limitations. First, the risk factors for CKD were assessed using maximal lymphocyte count because maximal lymphocyte count was strongly correlated with lymphocytosis during the study period [13]. Therefore, we consider that maximal lymphocyte count is potentially associated with lymphocytosis. Second, this analysis was conducted only during the periods of exposure to dasatinib. Indeed, TKIs are switched for various reasons, such as resistance and/or intolerance to prior therapy. Obviously, further studies including the period received other TKIs are required. In addition, while there is a favorable safety profile for kidney function in patients treated with dasatinib compared with imatinib [21], none of the 20 patients who received other TKI such as imatinib, developed CKD. In this regard, median age (40 years) at the initiation of other TKI treatments prior to dasatinib was consistent with a previous report, comprising patients whose median age 42 years did not develop CKD [22]. In addition, since patients with CKD at baseline were excluded, we considered that the administration of other TKIs prior to dasatinib did not influence the development of CKD. Lastly, this study consisted of the small number of patients as well as shorter follow-up duration. In particular, the follow-up duration (median 2.3 years) was shorter than that in a previous report (approximately 4 years) [4]. Therefore, we cannot depict robust conclusions. Despite these limitations, our results suggest the possibility that lower lymphocyte counts during dasatinib treatment are associated with the development of CKD, and in contrast, higher lymphocyte count may lead to the prevention of CKD progression. Therefore, monitoring kidney function is indispensable for dasatinib treatment.

In conclusion, the development of CKD during dasatinib therapy was associated with lower lymphocyte counts. Therefore, the higher levels of lymphocytes induced by dasatinib treatment may prevent CKD progression.

## Data Availability

The data that support the findings of this study are available from the corresponding author upon reasonable request.
